# Clinical and Radiological Characterization of an Infant with Caudal Regression Syndrome Type III

**DOI:** 10.1155/2020/8827281

**Published:** 2020-10-26

**Authors:** Kavinda Dayasiri, V. Thadchanamoorthy, Kaushika Thudugala, Aruni Ranaweera, N. Parthipan

**Affiliations:** ^1^Department of Paediatrics, Base Hospital Mahaoya, Maha Oya, Sri Lanka; ^2^Faculty of Health Care Science, Eastern University, Chenkalady, Sri Lanka; ^3^Department of Radiology, Base Hospital Mahaoya, Maha Oya, Sri Lanka

## Abstract

Caudal regression syndrome is a rare disorder of developmental failure of lumbosacral vertebra and corresponding spinal cord during notochord formation. The severity varies from absent coccyx to complete absence of lumbosacral vertebra and caudal spinal cord. Both genetic and environmental factors are believed to play roles in aetiopathogenesis of caudal regression. The authors report a two-month-old child born to a diabetic mother, in whom the diagnosis of caudal regression syndrome type III was confirmed based on clinical and radiological characteristics. The child was managed by the multidisciplinary team to continue supportive care and screen and monitor for long-term complications. The long-term prognosis for mobility was less favourable given the presence of bilateral hip dysplasia and involvement of lumbar vertebra in addition to sacral agenesis.

## 1. Introduction

Caudal regression syndrome is a rare congenital anomaly, which has an incidence of approximately 1 per 100,000 newborns [1]. The clinical features of this condition were first reported by Geoffroy Saint-Hilaire and Hohl in 1852 [2]. However, it was first proposed as a distinct clinical entity by Duhamel in 1960 [3]. Although the precise aetiology is unknown, poorly controlled maternal diabetes, vascular hypoperfusion, and genetic factors are thought to be contributory factors [4].

Caudal regression syndrome characteristically involves lumbosacral vertebra and corresponding segments of the spinal cord, which innervate structures in the pelvis and lower limbs due to a developmental failure [5]. Caudal agenesis is associated with malformations such as renal and ureter agenesis, duplex ureters, neuropathic bladder, vesicoureteric reflux, hydronephrosis, anorectal atresia, and imperforated anus [6]. Severe variants are often associated with cardiovascular, pulmonary, gastrointestinal, and musculoskeletal abnormalities. The authors report a two-month-old child, in whom the diagnosis of caudal regression syndrome type III was confirmed based on clinical and radiological characteristics.

## 2. Case Presentation

A two-month-old child was referred for evaluation of multiple congenital abnormalities. He was third born to nonconsanguineous parents with two healthy elder siblings. Antenatal period was uncomplicated apart from gestational diabetes, which was not controlled during the second trimester and before the diagnosis was made. Mother was on folic acid supplementation as recommended, and there was no history of radiation exposure or ingestion of long-term medications apart from being on antidiabetic medications.

**Figure 1:**
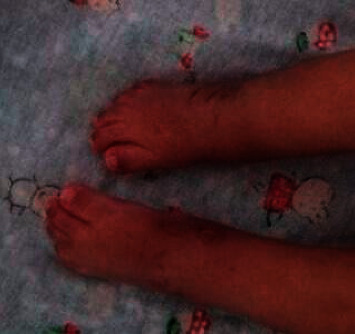
Bilateral congenital talipes equinovarus deformity.

**Figure 2:**
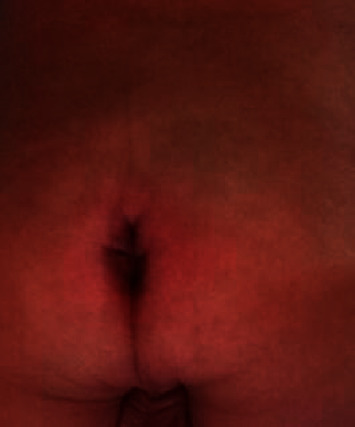
Displaced, patulous anus with flat, dimpled buttocks and no evidence of meningocele/meningomyelocele.

**Figure 3:**
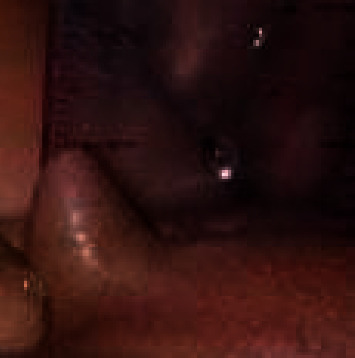
Micropenis (stretched penile length at 2 months of age: 1 cm).

He had been having constant leakage of urine and stools since birth. Growth was age appropriate. Physical examination revealed a narrow pelvis, bilateral knee flexion contractures, bilateral leg muscle atrophy, and bilateral congenital talipes equinovarus deformity with diminished ankle joint creases. He had clinical evidence of bilateral hip dysplasia with positive Ortolani's and Barlow's test. Other abnormalities included micropenis (stretched penile length: 1 cm) and displaced patulous anus with tiny pressure sores and flat, dimpled buttocks. Figures 1–3 show bilateral congenital talipes equinovarus deformity, appearance of the buttocks, and micropenis noticed in the reported child.

**Figure 4:**
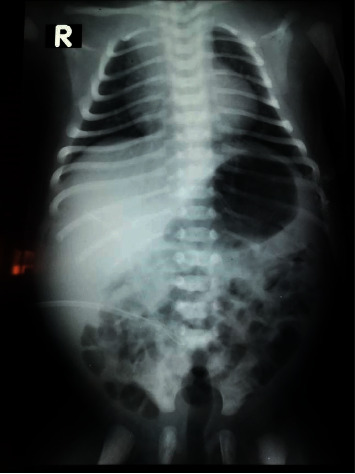
X-ray spine anteroposterior view: absent L5 vertebra and sacrum and bilateral iliac bones attaching to L4 vertebra.

**Figure 5:**
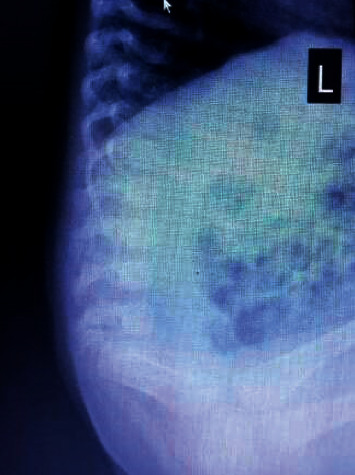
X-ray spine lateral view: absent L5 vertebra and sacrum.

**Figure 6:**
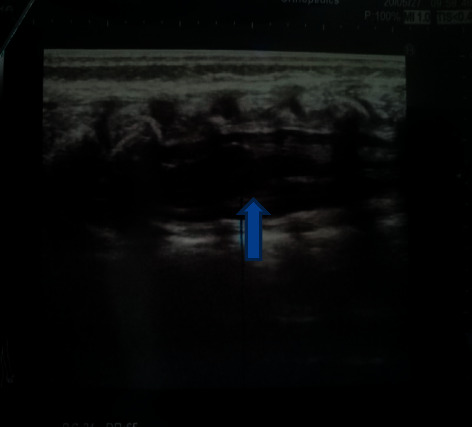
Ultrasound view: abrupt termination of spinal cord at the L1 vertebra level (arrow) and thickened conus medullaris.

**Figure 7:**
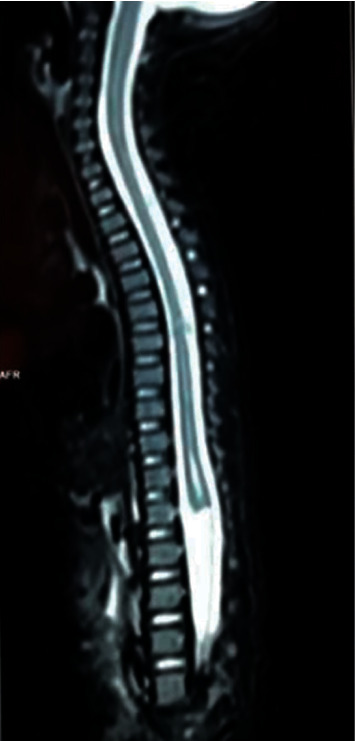
MRI spine: abrupt termination of spinal cord at the L1 vertebra level, syrinx involving D11-L1 measuring 17 mm in length and 1 mm in diameter.

He was investigated with X-ray of whole/spine and ultrasound of lumbosacral spine. Ultrasound revealed complete agenesis of the sacrum and L5 vertebra. The iliac bones were articulated with L4 vertebral body. The cord had terminated abruptly at the L1 level. Thickened conus medullaris was seen. There was no evidence of either meningomyelocele or meningocele. Ultrasound of hips revealed bilateral hip dysplasia with shallow acetabula. Overall, the findings were in keeping with type III caudal regression syndrome. Figures 4 and 5 show anteroposterior and lateral views of lumbosacral spine, and Figure 6 shows ultrasound appearance of abrupt termination of spinal cord at the L1 level.  Figure 7 demonstrates abrupt termination of spinal cord at the L1 vertebra level and a syrinx involving lower thoracic spine.

Abdominopelvic ultrasound showed no evidence of renal agenesis, neurogenic bladder, vesicoureteric reflux, and bowel malrotation. Serum creatinine was within the normal range. 2D echocardiogram was normal. MRI brain was also normal.

Parents were counseled regarding long-term prognosis and available supportive treatment options. Long-term follow-up was arranged with the general paediatrician to monitor for growth, development, and screen for long-term health issues including the neurogenic bladder. Early interventions were commenced to ensure sitting stability; however, the probability of ambulation was low as he had bilateral hip dysplasia, leg muscle wasting, and fixed knee contractures resulting from an advanced lumbosacral vertebral defect. The child was referred to orthopedic surgeon to discuss treatment options to improve pelvic stability. Follow-up was also arranged with the paediatric neurologist.

## 3. Discussion

Caudal regression syndrome is characterised by failure of development of lumbosacral vertebra and caudal spinal cord, and the severity could range from absent coccyx to total absence of lumbar and sacral vertebra. The aetiology is complex with implication of both environmental and genetic factors in aetiopathogenesis [7]. The underlying defect is failure of notochord formation during the gastrulation phase of embryogenesis [8]. The mother of this child had undetected and hence uncontrolled diabetes during the second trimester of pregnancy. Hyperglycemia is considered to be a strong risk factor for caudal regression syndrome. This is due to its negative impact on oxidative stress and DNA structure of which both can increase the risk of malformations in the fetus [9].

Clinical features of sirenomelia and VACTERL (vertebral defects, anal atresia, cardiac defects, tracheoesophageal fistula, renal anomalies, and limb anomalies) association overlap with clinical features of caudal regression syndrome [10]. Cardiac evaluation of this child was normal, and there were no tracheaesophageal malformations. Similarly, there was no fusion of soft tissues of lower limbs as seen in reported children with sirenomelia [11].

The current classification of caudal regression syndrome to four distinct types was proposed by Renshaw in 1978 based on the type of vertebral defect and nature of attachment of iliac bones to lowest vertebra [12]. Type I is characterized by either partial or complete unilateral sacral agenesis. Type II is the most common form, and in addition to partial sacral agenesis, there is a bilaterally symmetrical defect between ilia and either normal or hypoplastic first sacral vertebra. Type III is characterised by total sacral agenesis and variable lumbar vertebral agenesis. The two iliac bones are attached to the sides of the lowest vertebra. Type IV is the worst type, and either fusion of iliac bones or iliac amphiarthrosis is seen in addition to features described in type III. High abrupt termination of spinal cord as seen in this child is considered to be a hall mark of caudal regression syndrome [13].

Type III caudal regression syndrome as proposed by Renshaw is rarely described in literature. Its clinical features are largely similar to type I caudal regression described in Pang's classification [5].  Table 1 illustrates previously reported children/fetuses that had radiological features of type III caudal regression syndrome.

Management of types III and IV caudal regression is supportive, and aim of rehabilitation is to improve functionality rather than correcting deformities. There are limited data on optimal correction of deformities by orthopedic interventions for children with types III and IV caudal regression. Multilevel pedicular screw fixation combined with spinopelvic fusion to improve stability and sagittal spine balance was associated with enhanced quality of life [18].

The severity of defect in vertebra and spinal cord and associated malformations determines long-term prognosis [20]. Caudal regression is associated with long-term neurological, orthopedic, and urologic complications. Although there were no associated malformations in this child, the long-term outcome for functionality is less favourable given he had both lumbar and complete sacral agenesis and bilaterally dysplastic hips.

## 4. Conclusion

Type III caudal regression syndrome is characterised by complete sacral agenesis, lumbar vertebral agenesis, and articulation of two separate iliac bones on either side of the lowest vertebra. High abrupt termination of spinal cord is a hall mark of caudal regression syndrome. It is important to rule out VACTERL association as a differential diagnosis given the involvement of multiple organ malformations in both conditions. Long-term rehabilitation in caudal regression types III and IV is aimed at improving functionality rather than correcting deformities.

## Figures and Tables

**Table 1:** Previously reported children/fetuses with radiological features of type III caudal regression syndrome.

	Associated clinical findings	Reported risk factors
Boruah et al. [14]	Triangular pelvis	Diabetes
Kumar et al. [15]	Bilateral grade V vesicoureteric reflux and situs inversus totalis	Diabetes
Zaw et al. [16]	—	Diabetes
Hashami et al. [17]	Partial agenesis of the corpus callosum and partial lobar holoprosencephaly	Diabetes
Vissarionov et al. [18]	Kyphosis, bilateral hip dysplasia	—
Balioglu et al. [19]	—	—

## Data Availability

All data used to support the findings of the study are available within the article.
